# New clinical trial designs in the era of precision medicine

**DOI:** 10.1002/1878-0261.12465

**Published:** 2019-02-22

**Authors:** Elena Garralda, Rodrigo Dienstmann, Alejandro Piris‐Giménez, Irene Braña, Jordi Rodon, Josep Tabernero

**Affiliations:** ^1^ Medical Oncology Department Vall d'Hebron University Hospital and Institute of Oncology (VHIO) Universitat Autònoma de Barcelona Spain; ^2^ Early Drug Development Unit (UITM) Vall d'Hebron Institute of Oncology (VHIO) Barcelona Spain; ^3^ Oncology Data Science (ODysSey) Group Vall d'Hebron Institute of Oncology (VHIO) Barcelona Spain; ^4^ MD Anderson Cancer Center Houston USA

**Keywords:** adaptive designs, Basket of Baskets, basket protocols, Cancer Core Europe, umbrella protocols

## Abstract

Cancer treatment has made significant strides towards the promise of personalized medicine. Recent scientific advances have shown that there are numerous genetic deregulations that are common in multiple cancer types, raising the possibility of developing drugs targeting those deregulations irrespective of the tumour type. Precision Cancer Medicine (PCM) was born out of accumulated evidence matching targeted agents with these tumour molecular deregulations. At the same time, the therapeutic armamentarium is rapidly increasing and the number of new drugs (including immune‐oncology agents) entering drug development continues to rise. These factors, added to strong collaboration with regulatory agencies, which have approved novel agents based on data obtained from phase 1/2 trials, have led to unprecedented evolution in the design of early‐stage clinical trials. Currently, we have seen rapid phase 1 dose‐escalation trials followed by remarkably large expansion cohorts, and are witnessing the emergence of new trials, such as adaptive studies with basket and umbrella designs aimed at optimizing the biomarker–drug co‐development process. Alongside the growing complexity of these clinical trials, new frameworks for stronger and faster collaboration between all stakeholders in drug development, including academic institutions and frameworks, clinicians, pharma companies and regulatory agencies, have been established. In this review article, we describe the main challenges and opportunities that these new trial designs may provide for a more efficient drug development process, which may ultimately help ensure that PCM becomes a reality for patients.

**Abbreviations**
BOB: Basket of Baskets
CCE: Cancer Core Europe
ctDNA: circulating tumour DNA
dMMR: defective mismatch repair
FDA: Food and Drug Administration
MSI: microsatellite instability
NCI: National Cancer Institute
NSCLC: non‐small‐cell lung cancer
NTRK: neurotrophin tropomyosin receptor kinase
PCM: Precision Cancer Medicine

## 1 Introduction

Over recent years, much progress has been made in rendering cancer treatments more precise. Scientific and technological advances have unmasked numerous genetic deregulations that are common across multiple cancer types. These discoveries have led to the development of drugs targeting driver gene alterations irrespective of their primary tumour location. We are consequently witnessing the ringing in of a new era characterized by considering tumours as genetic diseases as opposed to tissue‐dependent processes. This is accelerating the pace from molecular aberration discovery to the approval of new therapies (Dienstmann *et al*., 2013a,b; Hierro *et al*., 2017; Hunter, 2016).

At the same time, during these last years the arrival of immune therapy to the oncology therapeutic armamentarium has marked a groundbreaking milestone for the treatment of cancer patients, and the number of immune‐oncology agents entering drug development continues to rise (Martin‐Liberal *et al*., 2017). These factors, added to the strong collaboration with the regulatory agencies, approving novel agents based on data obtained from phase 1/2 trials, have led to an unprecedented evolution in the design of early‐stage clinical trials (Bui and Kummar, 2018). In this regard, the US Food and Drug Administration (FDA) has approved 10 anticancer drugs matched to companion diagnostic biomarkers based on data obtained from nonrandomized trials in the last 2 years. This evolution is mainly driven by the desire to facilitate patients’ access to drugs with promising activity from the early stages of development and is also a consequence of pharmaceutical companies striving to obtain rapid regulatory approval of their anticancer medicines.

The traditional drug development track, where drugs were evaluated for safety in phase 1, early signs of efficacy in phase 2 and finally evaluated against standard therapy in a randomized phase 3 clinical trial, has gradually faded out. Currently, we are facing rapid phase 1 dose‐escalation trials followed by strikingly large expansion cohorts and the emergence of new trials such as adaptive studies with basket and umbrella designs aimed at optimizing the biomarker–drug co‐development process. In parallel, with the growing complexity of clinical trials, new frameworks for stronger and faster collaboration between all stakeholders in drug development, including clinicians, pharma companies and regulatory agencies, have been established (Harrington *et al*., 2017; Weber *et al*., 2017).

## 2 Precision medicine trials for cancer

With the development and clinical use of molecularly targeted agents, it became clear that only selected patient populations would derive benefit from these therapies. Precision Cancer Medicine (PCM) was born out of the accumulated evidence on matching targeted agents with tumour molecular aberrations (Dienstmann *et al*., 2015; Hoelder *et al*., 2012). Those drugs designed to interact with a specific target, and especially those using predictive biomarkers, showed the highest relative improvement in response rate and survival (Ocana *et al*., 2013). Current knowledge generated from large‐scale collaborative sequencing projects, such as the Cancer Genome Atlas and the International Cancer Genome Consortium, in addition to publicly available resources such as the cBioPortal for Cancer Genomics and the Catalogue of Somatic Mutations in Cancer, has facilitated our understanding of the genetic interpatient tumour heterogeneity in multiple cancers subtypes (Dienstmann *et al*., 2013a). Most druggable genomic aberrations are present only in small‐to‐moderate proportions of patients, further emphasizing multicentre collaboration in early drug development as critical for successful clinical trial enrolment (Dienstmann *et al*., 2015).

In addition, recent studies have also described striking intrapatient intratumour heterogeneity and how clonal evolution under treatment pressure may represent major obstacles in PCM, questioning the value of a single‐needle biopsy or surgical excision to accurately capture the complete genomic landscape of a patient's cancer (Bedard *et al*., 2013; Hunter, 2016). Nevertheless, we believe that the described heterogeneity in genomic profiles particularly applies to bystander mutations and that true tumour‐driving events are usually present in the majority of subclones from the primary tumour as well as the metastatic lesions (Yap *et al*., 2012). Therefore, regimens targeting driver genomic alterations with high variant frequencies are expected to provide substantial tumour responses. Since clinical responses to targeted agents are consistently abrogated by the development of drug resistance, we consider repeated tumour biopsies of progressing lesions and/or characterization of circulating markers (tumour cells, tumour DNA) to be a key component of patients’ care, allowing the identification of mechanisms of resistance as well as potentially guiding alternative treatment options with experimental agents.

In terms of clinical trials incorporating biomarkers, an essential element that should be factored in is the turnaround time for test results, particularly with tumour clinical next‐generation sequencing, for patients undergoing molecular profiling. This is especially important in the metastatic setting when treatment decisions have to be made within a short time frame.

As an alternative to the traditional approach of centralized biomarker analysis prior to evaluating the inclusion of a patient in a trial, multiple academic centres have adopted a different strategy consisting of local prescreening at academic institutions while patients are still receiving standard treatment for advanced disease. While this approach is time and tissue saving and increases the chances of patient recruitment in early clinical trials, the financial burden of prescreening tests is transferred from trial sponsors to healthcare providers and academic institutions (Dienstmann *et al*., 2015; Rodón *et al*., 2012).

In order to efficiently and dynamically incorporate genomic data and assess the value of matching profiled patients whose tumours harbour unique genomic alterations to specific interventions or targeted therapies, clinical trials design for cancer diagnostics and therapeutics must take these rate‐limiting steps into consideration.

### 2.1 Adaptive studies

A clinical study with adaptive design is defined as one that includes planned opportunities to modify one or more specified elements of the study design and hypothesis based on data analysis (usually intermediate findings) of the study subjects. Research into the accumulated data is carried out within the study at specific, prospectively planned time‐points, and can be performed in a completely blind or a nonblind way. The term prospective refers to the fact that the change was preplanned (and the details specified) before the data were examined in a nonblinded manner (CHMP/EWP/2459/02, 2007). The final objective of adaptive designs is to learn from the accumulated data and apply what has been observed as soon as possible. The modifications to the study design that can be planned in the written protocol cover a broad range of possibilities. Examples include study eligibility criteria, proportion of randomization, addition of new treatments and sample size of the study (Gallo *et al*., 2006).

Adaptive design trials have been shown to increase the efficiency of traditional clinical trials by facilitating the selection of the dose, reducing the number of patients exposed to ineffective or potentially toxic doses, aiding the precise calculation of sample size and reducing the duration and costs of clinical development. These new designs in early drug development enable the integration of preclinical data, the incorporation of information beyond the traditional dose‐limiting toxicity period, findings from other trials and emerging safety data, thereby increasing the likelihood of accurately determining any benefit of a new treatment and complying more quickly with regulatory requirements for efficacy and safety (Harrington *et al*., 2017).

In this regard, the formal reasons for stopping the study in an intermediate analysis could be: (a) safety (if one of the interventions involves many adverse events); (b) efficacy (if it shows efficacy of one of the interventions); or (c) futility (if the objectives are not achievable or unlikely to be achieved with a statistical significance). On the other hand, the sponsor may have the option of responding to intermediate data on safety and efficacy in various ways, such as narrowing the trial approach (e.g. elimination of one or more treatment groups based on futility criteria) or increasing the number of participants (e.g. If the data available at the time of the review do not allow for a clear decision between utility and futility, the enrolment of participants might be extended to one or more treatment groups beyond the initially intended sample). Cost reduction is achieved through the early identification of successful groups, leaving unnecessary treatment groups out of the equation or determining effective dosing regimens more quickly (Menis *et al*., 2014; Korn and Freidlin, 2017)

Well‐known examples of adaptive measures in clinical trials include early stopping rules in the instances of a lack of efficacy or unacceptable toxicity and altering doses or schedules of drugs in order to improve the benefit–toxicity profile. More recently, novel adaptation strategies have been proposed. In the adaptive accrual design, after the initial ‘learning phase’, the ratio of patients randomly assigned to the experimental arm vs the control arm changes from the standard 1 : 1 to increase the proportion of patients randomized to the arm that is performing better, which augments the statistical power to detect a relevant magnitude of clinical benefit.

The Biomarker‐integrated Approaches of Targeted Therapy for Lung Cancer Elimination 2 (BATTLE‐2 study), for example, is a biomarker‐based and biopsy‐mandatory prospective trial to guide treatment of heavily pretreated metastatic non‐small‐cell lung cancer (NSCLC) patients (NCT01248247). In the ‘adaptive phase’, randomization to different drugs or combinations is weighted based on mutation profile results generated in real time. A similar framework can be applied to studies assessing the predictive value of gene expression signatures. Instead of using a fixed model – built on the training data only – adaptive strategies use the information on patients enrolled earlier in the testing set to continuously update the model and refine accrual throughout the entire study (Dienstmann *et al*., 2015; Xiao *et al*., 2014).

Adaptive models increase the weights of good predictors and decrease the weights of unstable predictors, improving the overall performance of the classifier and selecting the ‘best’‐matched therapy to current patients’ characteristics. These algorithms may facilitate the use of molecular signatures to predict the clinical outcomes of patients in prospective clinical studies.

In the realm of precision medicine, enrichment trials with adaptive designs where predictive algorithms incorporating prior knowledge (based on *in silico* models of drug sensitivity, *ex vivo* experiments, preclinical or early clinical data) are used to guide the best‐matched targeted therapy in particular settings have been envisioned. This may be useful when multiple druggable alterations are identified in a patient's tumour sample and more than one agent is available for testing; and when one driver genomic event is identified, and the investigator has to select among various drugs with overlapping mechanisms of action (targeting the same driver event) but with different potency/activity according to coexisting genomic alterations. These ‘machine‐learning predictive models’ can complement molecular tumour boards efforts to identify the ‘best guess’ (Pemovska *et al*., 2013).

Both the EMA (2007) and the FDA have already recognized the validity of clinical trials with adaptive characteristics as a viable alternative strategy for both pivotal and early trials in the regulatory environment of pharmacological development (9). However, regulatory agencies are still reluctant in some cases to consider adaptive designs, as the results can be more difficult to interpret. One of the main concerns is the control of the type I error rate as well as the fact that adaptive measures may introduce bias (Bauer *et al*., 2016; Menis *et al*., 2014). Another important challenge of this type of studies is providing the information to the patient in a sufficiently precise but at the same time comprehensible way. These studies have complex designs, with several cohorts and one or several drugs under investigation. Above all, they can vary over time, which increases the uncertainty of the trial design and makes it very difficult to explain to the patient. To address this challenge, the trials will have different informed consents depending on the specific cohort (Korn and Freidlin, 2017).

### 2.2 Umbrella protocols

An umbrella trial is a master protocol for which the patient's eligibility is defined by the presence of a tumour type that is substratified according to specific molecular alterations matched to different anticancer therapies (Woodcock and LaVange, 2017).

Several Umbrella Protocols, in which patients were stratified by potential molecular biomarker and assigned to matched therapies, were initiated to evaluate the role of Precision Medicine in certain tumour types, such as the I‐SPY1/2 (Carey and Winer, 2016; Das and Lo, 2017) in breast cancer, the BATTLE 1/2 (Papadimitrakopoulou *et al*., 2016) in lung cancer, or the FOCUS‐4 (Adams *et al*., 2018) and MoTriColor (2015) in colorectal cancer (H2020 grant agreement no. 635342). Some studies tested or are testing this framework across multiple solid tumours, such as MOSCATO (Massard *et al*., 2017) or National Cancer Institute (NCI)‐MATCH trials (Mullard, 2015).

In some studies, such as MOSCATO, patients had limited access to a set of matched therapies, while others including I‐SPY and NCI‐MATCH have overcome this limitation by building networks and efficient partnerships with the pharmaceutical industry. Despite strong collaborations, some studies have been limited by the suboptimal biomarkers and matched drugs used including the BATTLE or the SHIVA trials (Adams *et al*., 2018; Le Tourneau *et al*., 2015). To deal with this limitation, the I‐SPY1/2 studies and the NCI‐MATCH have successfully implemented adaptive designs allowing the addition of new arms as new knowledge became publicly available. Most studies have remained fixed to their initial treatment algorithms, sometimes outdated at the time of study closure. Also, there was no flexibility to integrate new technologies that might be of interest, RNA‐based multiplexed assays for the detection of fusion events.

The I‐SPY1/2 studies and the NCI‐MATCH have successfully implemented flexible designs allowing for the addition of new arms as new data become publicly available, while others have remained fixed to their initial treatment algorithms that could be outdated by the time they close. In most of these studies, tumour molecular characterization is based on DNA analysis platforms, with little flexibility to integrate other technologies that might be of interest, such as RNA‐based multiplexed assays for the detection of fusion events and gene signatures that define unique portraits of tumours. An example of the latest approach is the MoTriColor EU H2020‐funded project, a set of molecularly guided trials with specific treatment strategies in patients with advanced newly molecular defined subtypes of colorectal (gene signature‐based) cancer (H2020 grant agreement no. 635342).

### 2.3 Basket trials

Basket trials include patients with different tumour types with a common molecular alteration who are treated with the same matched therapy (Carey and Winer, 2016; Redig and Jänne, 2015). They constitute a histology‐agnostic approach to evaluate targeted agents in molecularly selected populations and can provide access to experimental therapies for patients across a wide range of tumour types, potentially including those rare tumours that would have not been studied in other clinical trials (Billingham *et al*., 2016). The first basket study design evaluated the efficacy of vemurafenib in solid tumours or haematological malignancies harbouring *BRAF* ^V600^ mutations (NCT0152497). This design evidenced the activity of vemurafenib in BRAF V600E mutant NSCLC and also showed activity in other tumour types such as ovarian cancer and certain central nervous system cancers (Hyman *et al*., 2015).

Limitations of this approach include the following: (a) the assumption that the same mutation might have the same impact regardless of histology; (b) the presence of variants of unknown significance whose function has not previously been evaluated; (c) an inherent focus on one single alteration (when nowadays multimarker analysis is standard); and (d) without a control arm, it can be difficult to differentiate predictive from prognostic value of a biomarker. One important caveat is the possibility of an insufficient representation of patients with certain tumour types that harbour the alteration of interest, leading to false‐negative conclusions. Therefore, basket trials should be stratified by histology, taking into consideration the reported frequencies of the genomic event (Bedard *et al*., 2013). This strategy may be even adapted to increase enrolment of patients with tumour types that demonstrate early signals of antitumour activity while excluding those lacking preliminary response. Furthermore, additional cohorts with different tumour types can be created, and patients with related molecular aberrations can also be enrolled – those with newly identified fusion genes known to activate the pathway and sensitize to the agent under investigation similarly to gene copy number alterations or mutations, for example (Dienstmann *et al*., 2015). Moreover, if other driver molecular alterations coexist, patients could be offered combination regimens targeting more than one single alteration.

The rarity of certain tumour–biomarker combinations makes it impossible to conduct randomized trials. As a consequence, basket trials have increasingly been used as a potential means of providing the clinical data necessary to support such a shift in treatment approach. On 23 May 2017, the FDA granted the accelerated approval of pembrolizumab for the treatment of adult and paediatric patients with unresectable or metastatic refractory tumours harbouring microsatellite instability/defective mismatch repair (MSI‐H/dMMR). The first FDA tissue‐agnostic approval became a reality, based on the tumour response rate and the durability of response seen in 149 patients with MSI‐H/dMMR tumours across the five uncontrolled, open‐label, single‐arm trials (Diaz *et al*., 2017). Response rates were comparable across tumours, with 36% in colorectal cancer vs 46% in 14 other cancer types.

Following the approval of pembrolizumab, another very recent milestone has revolutionized the drug development field, with the approval of larotrectinib on November 2018 for the treatment of adult and paediatric neurotrophin tropomyosin receptor kinase (NTRK) rearranged tumours. Patients with NTRK fusion‐positive tumours were enrolled into the adult phase 1, the paediatric SCOUT phase 1/2, or the NAVIGATE ‘basket’ phase 2 trials evaluating larotrectinib, a first‐in‐class pan‐TRK inhibitor (Drilon *et al*., 2018). The expanded 122‐patient integrated dataset showed an overall response rate of 81% (95% CI, 72–88). Remarkably, 84% of the responding patients and 73% of all patients remained on larotrectinib or underwent surgery with curative intent. Strikingly, larotrectinib demonstrated clinical benefit regardless of tumour type, NTRK gene, fusion partner or age of the patient (Tan *et al*., 2018). Both of these approvals inaugurate a new area, with multiple new agents, such as RET inhibitors, following this new path (Garber, 2018; Subbiah *et al*., 2018).

## 3 The Basket of Baskets study: an example of EU collaborative basket framework with multimodular design

The Basket of Baskets (BoB) study is the spearhead Program of the Cancer Core Europe (CCE) (Eggermont *et al*., 2014). Its overall goal is to evaluate the antitumour activity of matched therapies in small CCE patient populations molecularly selected using a novel study design in an international multicentre (basket) approach.

**Figure 1:**
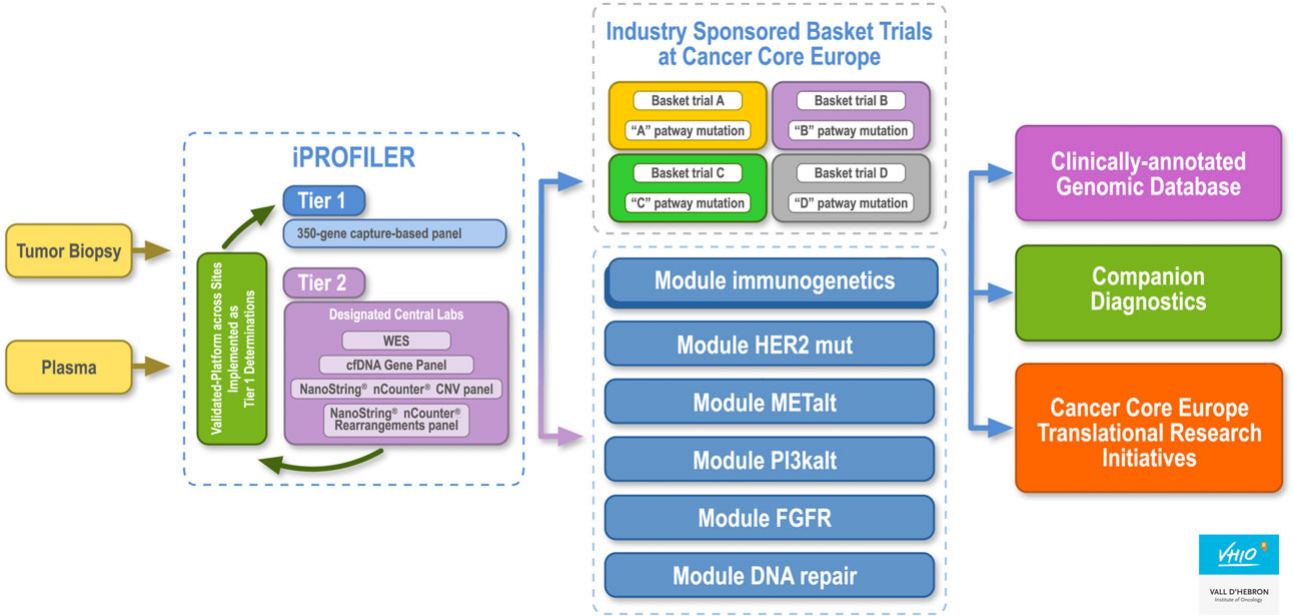
Design of the BoB platform sponsored by CCE.

The study consists of two parts: (a) I‐Profiler will allow the molecular characterization of tumours from patients with metastatic or recurrent solid tumours using a new profiling tool and select the most suitable treatment for these patients; and (b) I‐Basket is a multimodular basket trial, with different cohorts for genomically selected populations. Pharmaceutical companies will sponsor some of the specific treatment cohorts and will also benefit from the established collaboration and profiling to perform Pharma‐sponsored trials (Fig. 1) (Calvo *et al*., 2018).

The BoB study is testing therapies in multiple disease settings/genetic contexts, encompassed by the development of companion diagnostics based on specific biomarkers in these genetic contexts, including circulating tumour DNA (ctDNA) analysis as a way to select patients for any of the tested drugs and thus increase the efficacy of treatments. A broader genetic analysis also facilitates the testing of the feasibility and value of whole exome sequencing in a clinical context, as well as the development of a database for the stratification of other patient populations that could benefit for drug repurposing.

The CCE setting has aligned seven academic sites with state‐of‐the‐art platforms for molecular patient selection, a critical mass of patients and a unique infrastructure including bioinformatics and translational research capabilities that can support clinical trials in small patient populations. The framework collaboration integrates standardized prescreening methods (including a 350‐genes panel in tier 1) and common standard operating procedures, contracts and budgets. Its design allows both the development of sponsor‐initiated trials and modular investigator‐initiated trials, providing flexibility for adding new arms with different molecular alterations. The consortium comprises investigators and industrial partners as a collaborative initiative to explore the antitumour activity of multiple drugs in many different genetic contexts, provider flexible tools for tumour analysis (from discovery to market), study implementation (from pilot studies to registration of new indications – repurposing) and translational research (mechanisms of resistance, heterogeneity). The design allows a cost‐effective use of the shared platforms and aims at dramatically accelerating new indications (repurposing) of the tested targeted therapies by providing clinical evidence of activity and validated companion diagnostics for use in confirmatory trials.

Basket of Baskets seeks to accomplish ambitious advances: (a) Advance logistics of genomically oriented clinical trials through a structure that incorporates novel concepts and approaches and facilitates cooperation between academic sites, diagnostic companies and pharma; and (b) implement this innovative clinical research strategy for the development of targeted therapies in numerous molecular alteration settings, driven by tight connectivity between experts in genetics, translational science and clinical research with the profiling tools to assess the clinical benefit of those targeted therapies. This will help bridge the existing gap between scientific discovery in basic and translational research and its application in a clinical research setting.

## 4 Conclusions

In this review, we present some of the complexities and recent advances of research in precision medicine. Concepts such as phase 1 expansion cohorts replacing phase 2 testing, regulatory approvals based on nonrandomized trials and tumour agnostic approvals are established concepts in the field of drug development. Challenges include technical limitations of molecular tests, logistical issues for patient accrual in clinical trials and critical, unsolved regulatory issues. Importantly, knowledge in genomics is steps ahead of our ability to therapeutically target tumours given that many mutations identified by sequencing are either linked to unapproved drugs or are not druggable by currently available therapies.

Genetic heterogeneity at intratumour and interpatient levels and clonal evolution of tumours over time remain among the major obstacles for precision medicine to materialize. Given the potential of genomic characterization of circulating tumour cells and ctDNA, we expect that these circulating blood biomarkers will be important for monitoring the emergence of treatment‐resistant clones under selective pressures and providing an efficient model of individualized therapy. Clinical trial strategies such as platform studies with adaptive designs, innovative endpoints and collaborative frameworks to interrogate the efficacy of drugs will be key to advancing precision medicine.

## Author contributions

All authors contributed to the writing and review of this article.

## Conflict of interest

EG declares Advisory role for Roche, Janssen, NeoMed Therapeutics, Boehringer and Ellipses Pharma; Speaker's fee from MSD and Direct Research Funding from Novartis. RD declares Advisory role for Roche and Novartis; Speaker's fee from Roche, Symphogen, Ipsen, Amgen and Sanofi and Direct Research Funding from Merck. AP‐G has no conflicts to declare. IB declares Advisory role for Orion Pharma; Speaker's fee from BMS, AstraZeneca and Merck Serono; and Financial Support for clinical trials or contracted research for AstraZeneca, BMS, Celgene, Gliknik, GSK, Janssen, KURA, MSD, Novartis, Orion Pharma and Pfizer. JR declares Advisory role for Novartis, Eli Lilly, Orion Pharmaceuticals, Servier Pharmaceuticals, Peptomyc, Merck Sharp & Dohme, Kelun Pharmaceutical/Klus Pharma, Spectrum Pharmaceuticals Inc, Pfizer, Roche Pharmaceuticals, and Ellipses Pharma; and Direct Research Funding from Bayer and Novartis; and serving as investigator in clinical trials with Spectrum Pharmaceuticals, Tocagen, Symphogen, BioAtla, Pfizer, GenMab, CytomX, Kelun‐Biotech, Takeda‐Millennium, Glaxo Smith Kline and Ipsen. JT declares Advisory role for Array Biopharma, AstraZeneca, Bayer, BeiGene, Boehringer Ingelheim, Chugai, Genentech, Inc., Genmab A/S, Halozyme, Imugene Limited, Inflection Biosciences Limited, Ipsen, Kura Oncology, Lilly, MSD, Menarini, Merck Serono, Merrimack, Merus, Molecular Partners, Novartis, Peptomyc, Pfizer, Pharmacyclics, ProteoDesign SL, Rafael Pharmaceuticals, F. Hoffmann‐La Roche Ltd, Sanofi, SeaGen, Seattle Genetics, Servier, Symphogen, Taiho, VCN Biosciences, Biocartis, Foundation Medicine, HalioDX SAS and Roche Diagnostics; and Financial Support for clinical trials or contracted research for Agendia BV, Amgen, Debiopharm, Janssen‐Cilag, Mologen AG, Novartis, Pharma Mar, Roche, Servier and Symphogen.
